# Delayed diagnosis of lymph node tuberculosis: time-honored importance of a thorough clinical examination, Cameroon

**DOI:** 10.11604/pamj.2015.21.38.6595

**Published:** 2015-05-19

**Authors:** Tanyi John Tanyi, Julius Atashili

**Affiliations:** 1 Family Health care Foundation Limbe, Limbe, Cameroon; 2 Faculty of Health Sciences, University of Buea, Buea, Cameroon

**Keywords:** History taking, physical examination, lymph node, diagnosis, resource-limited settings

## Abstract

History taking and physical examination is the cornerstone of medical diagnosis as will lead to correct diagnosis 90% of the time. We report a case of a 30-year-old black African man with lymph node tuberculosis diagnosed one year and six months later after onset of symptoms and signs. Clinicians especially those in resource-limited settings should go in for thorough history taking and complete physical examination which is the basis for correct clinical diagnosis, will provide valuable guide in deciding which tests to order and thus laboratory tests done for confirmatory purposes and also, has a cost-effective benefit for the patient.

## Introduction

Physical examination is the process of evaluating objective anatomic findings through the use of observation, palpation, percussion, and auscultation. The information obtained must be thoughtfully integrated with the patient´s history [1]. For centuries, doctors diagnosed illness using their own senses, by poking, prodding, looking, listening. From these observations, a skilled doctor can make amazingly accurate inferences about what ails the patient [2]. With the advent of modern complex technological tools as an aid to clinical diagnosis, clinicians are drifting from the rich and irreplaceable place of thorough history taking and complete physical examination; as a result, many doctors are abbreviating the time-honored bed side physical exam - or even skipping it all together [2–5].

## Patient and observation

We report a case of a 30-year-old man who presented electively in our clinic with a history of intermittent high grade fever and right lower quadrant non-radiating abdominal pain for 1 year + 6 months duration, for which he consulted and treated in 5 different health facilities with no favourable evolution. On review of his systems; presence of non-productive cough, intermittent night sweat, loss of appetite, nausea but neither vomiting nor altered bowel habit. His past history is remarkable for appendicectomy 10 months ago after he consulted for similar complaints. Also, there is a history of contact with relative diagnose with pulmonary Tuberculosis (TB) 2 years ago. On examination; our patient was normotensive (BP=110\70mmHg) febrile to touch (Temp=38°c), lost 10% body weight within 6 months, ill-looking, asthenic with moderately pink conjunctivae and anicteric sclera. Head and neck exam revealed palpable submental, right and left anterior and posterior superficial cervical lymph nodes and right and left axillary lymph nodes. Nodes were mobile, non-tender with the left cervical node having the largest diameter of 4cm. Presence of fine inspiratory crackles on chest examination. Abdominal examination revealed grid-iron incision mark and mild tenderness around the right lower abdominal quadrant. A working diagnosis of Mesenteric adenitis + Lymph node Tuberculosis was made. A Full Blood Count (microcytic hypochromic anaemia with Hb=11g\dl, lymphocytosis), Chest X-Ray (showed parahila nodes), normal abdominal ultrasound, lymph node biopsy and histopathology (showed granulomas with langham giant cells and central caseation). Patient was then placed on a 6 month Anti-TB regiment under Direct Observational Therapy. Two weeks after onset of treatment evolution was favourable with normal body temperature and gain of appetite. Three months later, patient gained 4kg body weight, no palpable submental and axillary lymph node; there is mark reduction in size of cervical lymph nodes. Patient is fine now, has no complaint and has resume work.

## Discussion

Physical examination is the process of evaluating objective anatomic findings through the use of observation, palpation, percussion, and auscultation. The information obtained must be thoughtfully integrated with the patient´s history and pathophysiology. Moreover, it is a unique situation in which both patient and physician understand that the interaction is intended to be diagnostic and therapeutic [1]. The dawn of the modern physical examination was in 1761, when Leopold Auenbrugger first described the technique of percussion in a treatise in Latin entitled *Inventum Novum* (or *New Invention*). Although Auenbrugger´s *New Invention* described the first modern physical examination technique, it did not describe an underlying philosophy of diagnosis. Clinical medicine in Auenbrugger´s time was a practice of Hippocratic or Galenic theory, and the physician´s task was to fit symptoms into an idealized theory [6, 7]. Physical examination follows after a keen listening to the patient history which may be complementary to the signs that will be elicited during the examination. In an article entitled *The History of the patient history since 1850*, Dr. Jonathan Gillis reveals the cognitive consequences of privileging sign over symptom, thus the place for complete physical examination: “Patient history remains important and become incorporated into physician examination as another set of elicited signs and medical observations, thus producing two histories: a superficial, chaotic story presented by the patient or parent and another deep, “true” history revealed by the skill of the physician. The theory and practice of this skill changed, but there is little change in the status of the patient history, which is considered a creation of the clinical encounter rather than an account of a patient´s story” [8]. A complete physical examination after a thorough history taking can contributes a great deal to having the correct diagnosis and also count down the number of laboratory tests requested for patient especially in a resource limited setting. Similar contributions to appreciate the type and number laboratory tests from correct diagnosis and differential diagnosis has been discussed many years ago by renown teachers and scholars of medicine especially by Drs. Robert Hutchinson and Harry Rainy [9, 10]. On a careful clinical examination of our patient we had a high index of suspicion for lymph node tuberculosis which has been found in literature, should be made in patient with cervical lymphadenopathy [11, 12]. Furthermore, we carried out an open lymph node biopsy (test readily available) which also, has been discussed in literature as the gold standard for the diagnosis of lymph node tuberculosis [11–14] where a definitive diagnosis was made and our patient later placed on anti-TB regiment under direct observational therapy.

## Conclusion

Obtaining a complete history and doing a thorough physical exam cannot be overstress as it remain beneficial to both the patient (has a cost-effective benefit) and clinician (is the basis for correct clinical diagnosis and will provide valuable guides in deciding which tests to order and thus laboratory tests done for confirmatory purposes). Also, it will go a long way to maintain the standards of good medical practice as performed by the fathers of medicine.
